# Microplastics at the ‘roof of the Americas’: widespread presence in snow on Mount Aconcagua, Central Andes, Argentina

**DOI:** 10.3389/ftox.2026.1878370

**Published:** 2026-10-08

**Authors:** Juan Manuel Ríos, Yoshifumi Horie, Mariano Hugo Masiokas, Diego Esteban Cargnelutti

**Affiliations:** 1 Laboratorio de Ecotoxicología, Instituto de Medicina y Biología Experimental de Cuyo (IMBECU, CCT-CONICET), Mendoza, Argentina; 2 Research Center for Inland Seas (KURCIS), Kobe University, Fukaeminami-machi, Higashinada- ku, Kobe, Japan; 3 Instituto Argentino de Nivología, Glaciología y Ciencias Ambientales, (IANIGLA), CCT- CONICET, Mendoza, Argentina; 4 Facultad de Ciencias Médicas, Universidad Nacional de Cuyo, Mendoza, Argentina

**Keywords:** Aconcagua Provincial Park, Central Andes, microplastics, Mount Aconcagua, plastic pollution, polyester fibers, snow

## Abstract

Microplastics (MPs) pollution has emerged as a pervasive contaminant in remote environments; however, its occurrence in high-altitude snow remains poorly constrained. Here, we provide baseline evidence of MP contamination in snow from four mountaineering camps on Mount Aconcagua (6,962 masl), the highest peak in the Americas and in the entire Western and Southern Hemispheres. Microplastics were identified using µFTIR-ATR spectroscopy. The mean MPs concentration was 38 MP L^-1^, with site-specific values of 79 ± 37, 27 ± 8.3, 40 ± 16, and 5 ± 1 MP L^-1^ at the Plaza de Mulas base camp, and at the Canada, Nido de Condores, and Berlin higher elevations camps, respectively. Significantly higher concentrations at the base camp highlight the influence of intense human activity on MP accumulation, even in these remote high-mountain settings. Fibers (<5 mm), predominantly blue, were the dominant shape (90%), and polymer analysis revealed polyester, polyethylene, and epoxy as the main constituents. These findings indicate that snow is an effective matrix for assessing atmospheric deposition of MPs in andine environments. The spatial distribution of MPs suggests that local emissions associated with mountaineering activities represent a key source, although long-range atmospheric transport cannot be excluded. Our study provides the first documented evidence of the pollution of MPs in this world-famous climbing area, underscoring the vulnerability of high-altitude ecosystems to plastic pollution. Given the ecological significance and increasing human pressure in the Aconcagua Provincial Park, these results highlight the urgent need for targeted waste management strategies and regulatory actions to mitigate MP inputs in this fragile mountain environment.

## Introduction

Microplastics (MPs) pollution has gained increasing attention in the last decade due to its ubiquity on a global scale. The presence of MPs has been reported in different matrices from remote and extreme environments such as the Arctic (Bergmann et al., 2019), Antarctica (Aves et al., 2022), the deepest area of the ocean (Jamieson et al., 2019), and the highest mountain in the world (Napper et al., 2020). Mountain environments serve as early warning systems for both climate change and the impact of human activities, and could therefore serve as sentinels of pollution (Schmeller et al., 2022). Although, there is a widespread belief that high mountains are pristine and pollution-free environments, in the last 5 years it has been shown that mountains are sinks of plastics debris and MPs that are exclusively of human origin (Liro et al., 2023; Wei et al., 2024). In mountains, these particles can be driven by atmospheric transport and deposition (Wei et al., 2024) and can also be released by local human activities (e.g., hiking, tourism, dumping sites). Few research reports the presence of MPs in highly visited peaks frequented by trekkers such as Mt. Everest (Napper et al., 2020), Mt. Fuji (Wang et al., 2023) and El Teide (Villanova-Solano et al., 2023). However, there is a gap in information on plastic pollution at the high peaks of the Central Andes, particularly Mount Aconcagua, which is a world-famous destination for climbers and trekkers.

Mount Aconcagua (32.65°S, 70.01°W, 6962 masl), Argentina, is the highest peak of the entire American continent and therefore is often called ‘The roof of the Americas’. This peak is also the highest mountain outside the Tibetan Plateau in Asia, making it a landmark destination for climbers all over the world. Nowadays, this mountain and the sites around it are facing a severe waste management crisis. Decades of climbing expeditions have left the mountain littered with plastic bags, food packages, cans, containers, abandoned tents and textile waste (Barros and Pickering, 2014; 2015; Barros and Marina Pickering, 2017). The seasonal accumulation of human waste presents additional, considerable environmental and public health risks, and effectively addressing this issue remains a persistent and complex challenge. The harsh conditions and high altitude of these locations (the Plaza de Mulas base camp is at 4350 masl) make waste removal difficult, leading to contamination of water sources and contributing to the spread of diseases. Researchers, climbers and trekkers are starting to call for more stringent measures and increased awareness to address this issue. In response, Argentine authorities are pursuing regulations grounded in scientific evidence to inform policy-making aimed at mitigating pollution and preserving the ecosystem of this iconic natural landmark.

Mountain snow is a crucial component of the Earth’s hydrological cycle. Snowmelt from mountain ranges feeds rivers, lakes, and groundwater systems, providing vital water supplies for many regions, and regulating the water cycle (Schmeller et al., 2022). The percentage contribution to the world’s readily available freshwater can vary depending on the study region. For example, in the western United States, approximately 75% of freshwater currently originates as snow in the Sierra Nevada, Rocky and Cascade mountains (Simpkins, 2018). In the central Andes of Argentina and Chile between 30° and 37°S, winter snow accumulation represents a critical water resource for cities, rural communities, and socio-economic activities such as irrigation, hydropower generation, industry, and recreation (Masiokas et al., 2006). Snowmelt during the warm season (October-March) modulates river discharges on both sides of the Andes and contributes significantly to aquifer recharge and natural ecosystems in this semi-arid region. Andean glaciers are also highly dependent on winter snow accumulation for their long-term survival (Masiokas et al., 2016). During the last 40 years, glaciers in the upper watershed of the Mendoza river (where Aconcagua is located) have contributed, on average, ca. 18% of the annual discharges (Toum et al., 2025).

The presence of MPs in snow from high-mountain areas of Himalayas (Napper et al., 2020; Zhang et al., 2021), Alps (Bergmann et al., 2019; Pastorino et al., 2021), Northern Andes (Cabrera et al., 2022), North America (Karapetrova et al., 2024), and Antarctica (Aves et al., 2022) evidence the atmospheric transport of these contaminants. Additionally, trekkers and climbers substantially contribute to the plastic footprint in snow along the trails and around camps used during expeditions, due to the use of technical clothing and waste dumps (Napper et al., 2020). The present study aimed to assess the presence of MPs in snow collected from four camps on Mount Aconcagua, varying in accessibility and human presence, and to evaluate the feasibility of using surface snow as a tool for monitoring MPs in high Andean mountains. Morphological characterization (size, shape and color) and the determination of their composition by attenuated total reflection Fourier-transform infrared (ATR-µFTIR) spectroscopy may help infer potential sources. To the best knowledge of the authors, this work constitutes the first scientific assessment of MPs in Aconcagua, and one of the very few articles in the literature dealing with the determination of MPs in the world’s most iconic high-altitude peaks.

## Materials and methods

### Study area and sampling

Mount Aconcagua is located in the Central Andes region of Argentina, at 32° 39ʼS and 69° 59ʼW and belongs to Aconcagua Provincial Park. Ecosystems surrounding Mt. Aconcagua including watersheds, glaciers and periglacial environments around this mountain (Alvarez et al., 2019). Aconcagua Park has been registering a significant increase in the total number of visits for several seasons. For example, from November 2022 to October 2023, around 440,000 visitors entered to Aconcagua Park (Gobierno, 2024). Due to security reasons, Aconcagua Provincial Park only receives visitors and hikers during the austral summer season, with access restricted during the winter. Summers are generally very dry, with only occasional snowstorms above 4000 masl in Mt. Aconcagua. According to Koeppen (1931) the climate of the Mt. Aconcagua at altitudes above 4,100 masl, is defined as Polar with eternal ice where the average monthly temperature never exceeds 0 °C and the ground remains frozen all year round. The average annual precipitation values ​​for the area reach 500 mm in the highest sectors, occurring mainly in the winter period in the form of snow (Alvarez et al., 2019).

Georeferenced sampling sites are shown in Table 1 and Figure 1. Snow samples were collected during a research expedition to Mt. Aconcagua at the onset of the austral summer. Sampling dates for each climbing camp are provided in Table 1. The expedition included four camps on Mt. Aconcagua: Plaza de Mulas Base Camp (Camp 1), Canada (Camp 2), Nido de Condores (Camp 3), and Berlin (Camp 4). At each site, an 8 m transect was established in a random direction. Four 500 mL aluminum bottles were placed at 2 m intervals along the transect (n = 4 per site). Snow collection was conducted against the wind to minimize cross-contamination with bare hands, then immediately sealed and stored in a sampling storage tent. Surface snow, defined as snow that had not yet melted since the previous winter season, was collected using a stainless steel spoon and transferred into the aluminum bottles. Both the bottles and the spoon had been previously cleaned in the laboratory with Milli-Q water (twice). No snowfall events occurred during the expedition. Upon return to the laboratory, all samples were stored at −20 °C until further processing. Due to logistical constraints, sample containers had to be transported by helicopter, which imposed strict limitations on weight and volume. Consequently, the number of sampling containers was restricted. In addition, all materials required for sampling above Plaza de Mulas Base Camp had to be carried manually uphill (empty containers) and downhill (filled containers), representing a substantial physical burden. Although the total number of samples analyzed was limited (n = 16), each site included replicates (n = 4), ensuring that the dataset remains reasonably representative of this remote and difficult-to-access environment.

**TABLE 1:** Georeferenced sites and sampling date of the settled snow* at each camp in Mount Aconcagua.

Site	Latitude (dd)	Longitude (dd)	Altitude (m.a.s.l.)	Collection date
Plaza de mulas base camp (camp 1)	−32.65111	70.06104	4340	01/17/2025
Canada camp (camp 2)	−32.64415	70.04104	5134	01/17/2025
Nido de condores camp (camp 3)	−32.63582	70.02864	5570	12/17/2024
Berlin camp (camp 4)	−32.63871	70.02183	5930	12/16/2024

* Snow that had not yet melted from the previous winter season.

**FIGURE 1:**
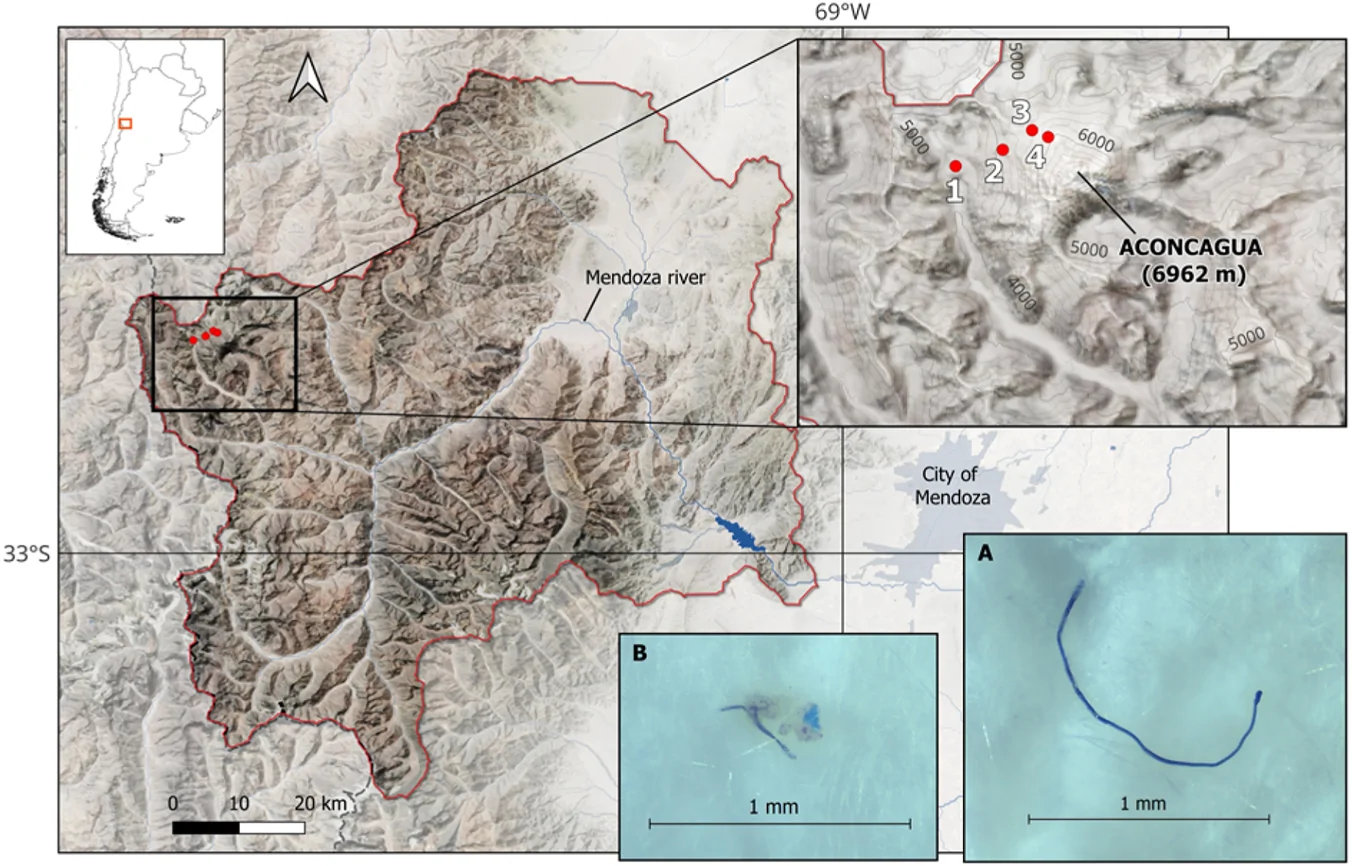
Map of sampling sites in Mt. Aconcagua: Plaza de Mulas Base Camp (1) Canada Camp (2), Nido de Condores (3), and Berlin (4). Red line denotes the upper Mendoza River basin in the Central Andes range in Argentina. Bottom left, some examples MPs detected in the snow of Mount Aconcagua: **(A)** blue fiber and **(B)** blue fragment and fiber.

### Laboratory analyses for characterization of microplastics in snow samples

Once in the laboratory, each sample was defrosted. After the snow melted at room temperature, the total volume of water to be filtered was measured to account for the value used in the calculation of MP concentrations. The abundance or concentration of MPs in snow was expressed as MPs L^-1^. All filtration steps were performed in a laminar flow cabinet (ScanLaf Fortuna, LaboGene). Cotton laboratory coats and clothes were worn to reduce contamination from synthetic textiles. Each sample was filtered using pre-rinsed glass equipment with Milli-Q water. The filtration setup was connected to a vacuum pump and polytetrafluoroethylene 10 µm filters (Omnipore 47 mm) were used. Each particle was photographed and classified as follows: A combination of visual inspection and spectroscopic analysis was employed to accurately identify, quantify, and characterize particles in the snow samples. Visual identification was performed using an Olympus SZX16 stereomicroscope equipped with a digital camera (Olympus DP74), at magnifications ranging from ×8 to ×10. Particles were classified according to size, shape (fibers or fragments), and color. Size measurements were based on the longest dimension; for fibers, only length was recorded. Polymer identification was determined using attenuated total reflection Fourier-transform infrared (ATR-µFTIR) spectroscopy (Shimadzu AIM-9000 coupled with an IRTracer-100). Spectra were acquired in absorbance mode as the average of 45 scans over the spectral range of 600–4000 cm^-1^, with a resolution of 8 cm^-1^. An aperture size of 25 × 25 µm was used for ATR-FTIR measurements. Polymer identification was achieved through automated comparison with an extensive spectral library using Shimadzu AIMsolution-9000 software, applying a similarity threshold of >70%. Particles identified by visual inspection that did not meet the criteria for microplastics (i.e., lacked a confirmed polymer identification) were excluded from further analysis.

#### Quality assurance and control

The identification of MPs in environmental samples, particularly fibers, is highly susceptible to cross-contamination due to their ubiquitous presence from textile wear and shedding, as well as their occurrence in laboratory materials and water sources (Vidal et al., 2021). To ensure accurate quantification and qualification of MPs, stringent contamination control measures were implemented throughout the analytical workflow, following established guidelines (e.g., Hermsen et al., 2018). Prior to laboratory work, all surfaces and materials were thoroughly cleaned, and work areas were routinely sterilized with 70% ethanol before and after each analysis. Air conditioning systems were turned off to reduce airborne particle transport, and all surfaces and materials (e.g., filtration funnels, Petri dishes, glass bottles, and vials) were covered with aluminum foil when not in use. Cotton lab coats were worn and plastic materials and gloves were never used to avoid cross-contamination. To monitor potential contamination, procedural blanks consisting of Milli-Q water placed in open Petri dishes near the working area were analyzed alongside the samples. These blanks were subjected to the same processing, observation, and identification procedures as the environmental samples. Any detected MPs in the blanks were carefully examined, and if polymer types matched those found in the samples, they were subtracted accordingly to correct for background contamination.

### Statistical analyses

Non-parametric statistics was used since raw data did not fit a normal distribution (Shapiro-Wilks W test) and a low number of snow samples from each study site (n = 4) were used. To test for differences in the concentrations of MPs among study sites (camps), Kruskal–Wallis *H* test (KW) followed by *a posteriori* multiple comparisons of mean ranks for all groups were applied. Statistical analysis was carried out using the InfoStat 2011 software (Di Rienzo et al., 2011). For all statistical comparisons, a value of *p* < 0.05 was considered significant.

## Results and discussion

### Microplastic abundance

The mean MP concentration in snow collected from Mt. Aconcagua was 38 MP L^-1^. Microplastic abundance varied among sampling sites (Table 2; Figure 2). At Plaza de Mulas base camp, mean concentrations reached 79 ± 37 MP L^-1^, whereas lower values were recorded at Canada (27 ± 8.3 MP L^-1^), Nido de Condores (40 ± 16 MP L^-1^), and Berlin (5 ± 1 MP L^-1^) camps (Table 2). Significant differences were observed among sampling sites (Kruskal–Wallis *H* = 9.75, *p* = 0.04) in median MP concentrations in snow. *A posteriori* analysis showed higher MP concentrations at Plaza de Mulas base camp (Figure 2) compared with Berlin camp. However, MP concentrations at base camp did not differ significantly from those at the other two sites (*p* > 0.05) (Figure 2). Plaza de Mulas base camp serves as a high-altitude acclimatization site for climbers before they begin their ascent to the summit. The majority of climbers spend several days (up to 7 days) at Plaza de Mulas base camp. During the climbing season, this site is characterized by the presence of numerous tents, water tanks, food and clothing stores, waste disposal areas, and frequent traffic of mules and helicopters (Figure 3). Therefore, it is not surprising that this site showed higher MP concentrations than other camps with lower anthropogenic influence, which provides a plausible explanation for the microplastic pattern observed at Mt. Aconcagua base camp. The high variability observed at the base camp, including one sample with comparatively elevated microplastic abundance, may reflect the influence of localized anthropogenic sources and episodic atmospheric deposition processes, which are expected to generate spatially heterogeneous microplastic distributions in high-altitude environments. The percentage contribution according to shape showed a predominance of fibers (90%) followed by fragments (10%) Supplementary Figure S1. In this study, fiber predominance is probably linked to the detachment of fibers from the climbing clothes, climbing gears and the windblown transport from hikers camps.

**TABLE 2:** Mean, median, and standard error (SE) for MP concentration (MPs L^-1^) found in snow collected at each camp (n = 4) on Mount Aconcagua.

Site	Mean	Median	SE
Plaza de mulas base camp (camp 1)	79	54	37
Canada (camp 2)	27	24	8.3
Nido de condores (camp 3)	40	29	16
Berlin (Camp 4)	5	4	1

**FIGURE 2:**
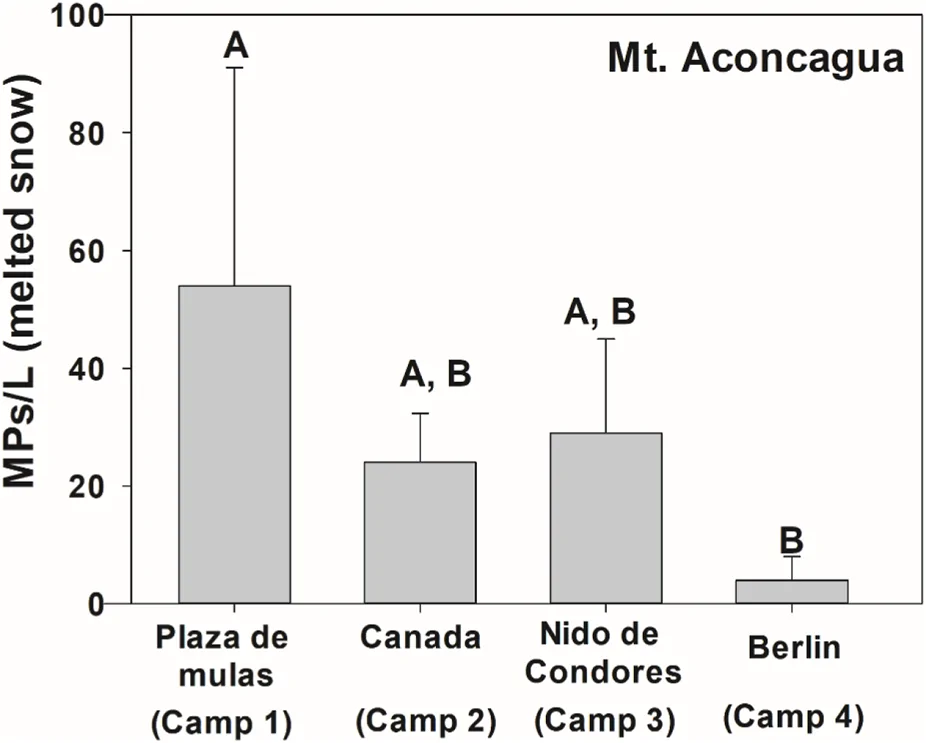
Microplastics concentration in snow collected in Mt Aconcagua: Plaza de Mulas Base Camp (Camp 1), Canada (Camp 2), Nido de Condores (Camp 3), and Berlin (Camp 4). Bars represent a median and the whiskers standard errors.

**FIGURE 3:**
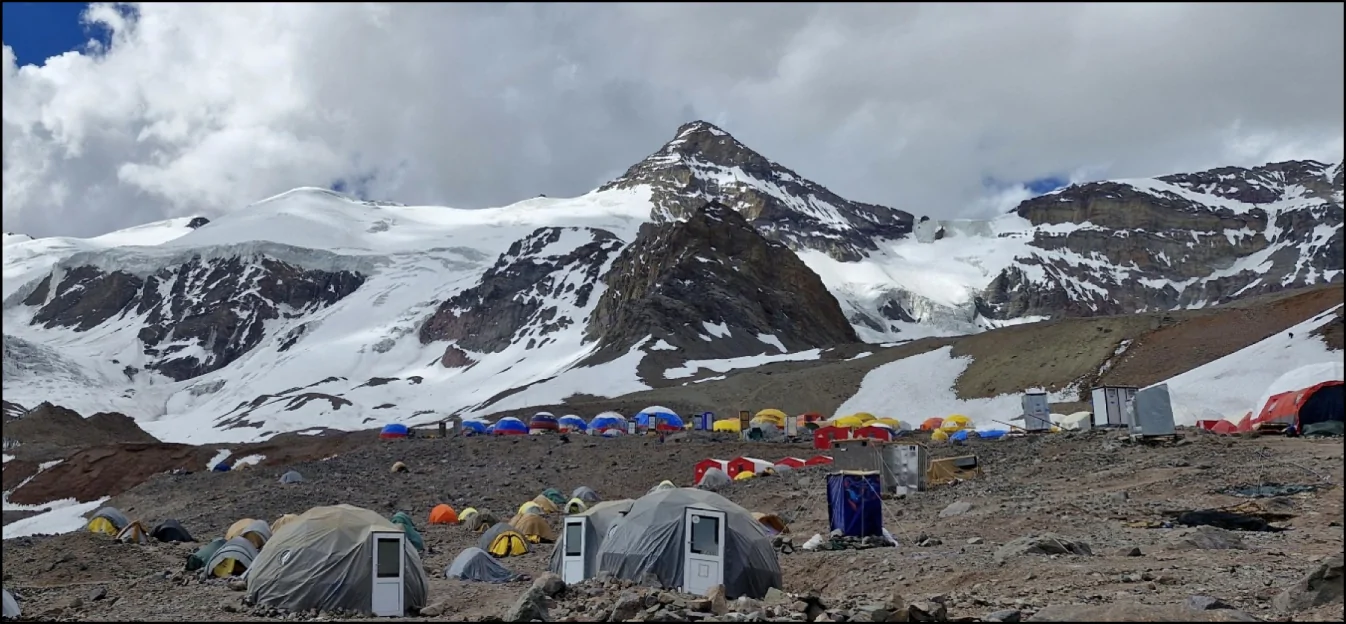
High-elevation camping tents that are made of waterproof polymeric materials. Photography taken at the Plaza de Mulas Base Camp (4340 masl), Mt. Aconcagua, Argentina.

This study provides a critical initial assessment of MP pollution on Mt. Aconcagua, reporting a mean snow concentration of 38 MP L^-1^ in snow. A comparable value of 30 MP L^-1^ in snow has been reported for Mount Everest, a highly visited peak popular with trekkers. Other studies conducted in climbing areas in the Teide National Park (Canary Islands, Spain) and in the Mount Conness (Sierra Nevada, California, United States) reported concentrations of 188 and 539 MP L^-1^ in snow, respectively (Villanova-Solano et al., 2023; Karapetrova et al., 2024). The mean concentration detected at Mount Terminillo in the Apennines was 74 MP L^-1^ in snow, and the authors suggest that this level could plausibly be attributed to its proximity to Rome (Pietrelli et al., 2025). Other studies conducted in Italian mountains reported very low MP concentrations in snow. For instance, Parolini et al. (2021) reported mean concentrations of 4.9, 2.5, 1.4, and 0.3 MP L^-1^ in snow respectively collected at Miserin, Deffeyes, Malatra, and Cuney mountain sites in the Western Italian Alps. Pastorino et al. (2021) reported a concentration of 0.1 MP L^-1^ in snow collected at the Carnic Alps. Regarding the Andes range, our results differ considerably from those reported by Cabrera et al. (2022) in the Northern Ecuadorian Andes. The authors argued that the average concentration of 131 MP L^-1^ in the surface snow from the remote Antisana glacier could plausibly be due to an atmospheric transport process across the Amazon basin (Cabrera et al., 2022).

### Microplastic characterization

Regarding the MP sizes, values of mean, median, standard error, and range according to each sampling site are shown in Supplementary Table S1. Only microplastic particles ≥0.2 mm were considered in this study. This lower size threshold was adopted as a methodological criterion to ensure consistent visual selection, manual handling, and reliable polymer identification by ATR-FTIR. Within the analytical workflow applied here, particles smaller than 0.2 mm could not be consistently isolated, manipulated, and placed in firm contact with the ATR crystal to obtain reproducible spectra. Therefore, particles <0.2 mm were excluded from the analysis to maintain analytical consistency. Although smaller particles are environmentally relevant and may contribute to long-range atmospheric transport, their characterization was beyond the scope of the present study.

The particle size distribution of the recovered microplastics (Supplementary Figure S2) varied among sampling sites. Overall, particles belonging to the smaller size classes (0.2–<1 mm) represented a substantial proportion of the recovered microplastics, although the predominant size class differed among sites. Larger particles (≥2 mm) were comparatively less frequent. This size distribution pattern is consistent with a possible contribution from synthetic fibers associated with technical clothing and outdoor equipment used by mountaineers and trekkers; however, atmospheric transport should not be excluded as an additional pathway contributing to microplastic deposition in this high-altitude environment. Further studies, particularly those including long-term temporal monitoring and atmospheric assessments, are required to better quantify the relative contributions of local sources and long-range atmospheric transport.

With respect to fiber color, the relative contribution of each color across sampling sites (each camp) is shown in Supplementary Figure S3. The seven detected fragments were white (57%) and blue (43%). Consistent with our findings, several other studies conducted in mountain environments have reported a higher abundance of blue microfibers in snow (Aves et al., 2022; Parolini et al., 2021; Pastorino et al., 2021; Villanova-Solano et al., 2023). Supplementary Table S2 summarizes the reported data on the concentration and characterization of MPs in snow in high altitude climbing areas as well as anthropized areas. Literature evidence indicates that microfibers are the most frequently reported type of microplastics in snow samples from high-altitude environments (Supplementary Table S2).

Attenuated total reflection micro–Fourier transform infrared spectroscopy was used to determine the polymer composition of microplastics in the snow samples. The identified polymers included polyester, polyethylene (PE), epoxy resins, polyarylate, polyvinyl chloride (PVC), and polystyrene-co-acrylamide (Figure 4). Regardless of the sampling site, the most abundant polymer was polyester (42%), followed by polyethylene (23%) and epoxy (23%), while polyarylate, PVC, and polystyrene-co-acrylamide were detected at lower frequencies (4% each) (Supplementary Figure S3). The predominance of polyester is consistent with its widespread use in textiles, suggesting that a substantial fraction of the detected microplastics may originate from synthetic fibers released from outdoor clothing and mountaineering gear (Napper et al., 2020). Polyethylene and PVC are commonly associated with packaging materials and consumer products, whereas epoxy resins are widely used in coatings and industrial applications (Aves et al., 2022). Although polymer identification alone does not allow definitive source attribution, it provides valuable insight into the potential origins and pathways of microplastics in high-altitude snow environments, as further discussed below.

**FIGURE 4:**
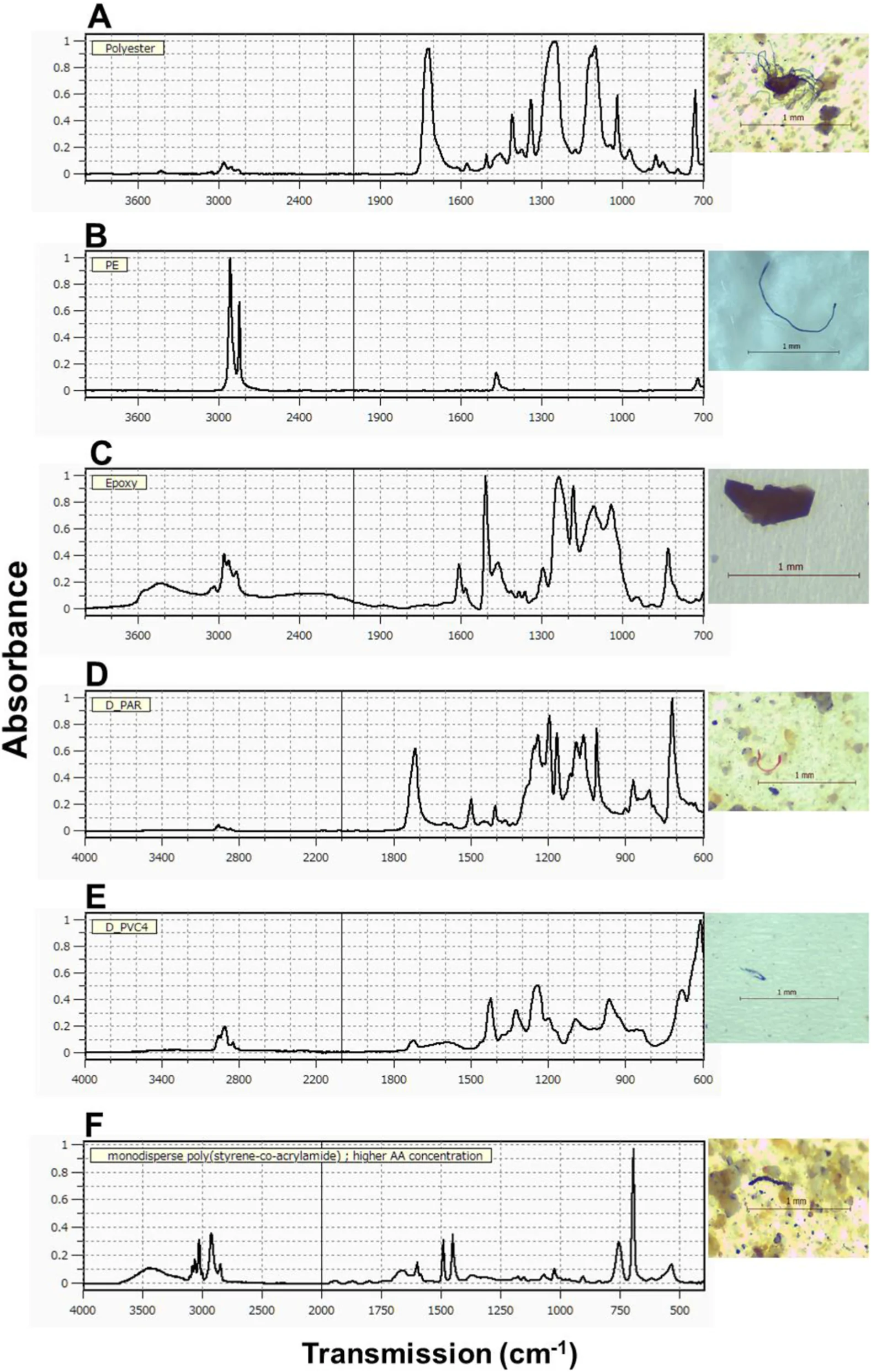
FTIR spectra obtained from the MPs present in the snow samples of the Mt. Aconcagua: **(A)** polyester, **(B)** polyethylene, **(C)** epoxy, **(D)** polyarylate, **(E)** polyvinylchloride, and **(F)** polystyrene-co-acrylamide.

### Possible plastic pollution sources and the implications for the Central Andes region

Several studies conducted in the Andes mountain range have reported the presence of MPs in different matrices. In the Northern Andes, MPs have been found in sediments (Canchari and Iannacone, 2023), snow (Cabrera et al., 2022), as well as in surface waters and edible fish (Loayza et al., 2022). In the Central Andes, MPs have been detected in both non-native and native fish species (Ríos et al., 2022; 2025), and native freshwater decapod crustaceans (Ríos, Manuscript submitted for publication). In the Southern Andes, MPs were reported in the surface waters of nine Patagonian lakes (Alfonso et al., 2020). These findings support the hypothesis of local, regional, and atmospheric transport of MPs along the Andes range. However, further research is needed to understand the dynamics and ecological impacts of these pollutants in the Andean region. For example, human activities have been linked to increased microplastic contamination in the Himalayas (Han et al., 2023). A plausible explanation is that MP loads, particularly polyester microfibers, found at popular climbing sites worldwide such as Mount Aconcagua, arise from both short- and long-distance transport, and in addition to that source, outdoor activities including tourism and hiking may increase the amount of MPs in the snow, as discussed below.

Given the increasing number of climbing expeditions and the rise in tourism activity in the Aconcagua Provincial Park, one plausible source of plastic pollution on Mount Aconcagua could be attributed to local transport. The tourist use recorded in 2011 was more than 6,000 trekkers and 5,600 pack animals, with visitor use concentrated mainly between November and March (Barros and Pickering, 2014; 2015; Barros and Marina Pickering, 2017; Dirección de Recursos Naturales, 2011). However, a decade later the number of visitors increased significantly to around 440,000 during the 2022–2023 climbing season (Gobierno de Mendoza, 2024).

It is also worth noting that the snowmelt from the Aconcagua area contributes directly to the Mendoza river and the northern oasis of the Mendoza province, the most important urban, agricultural and industrial center in central-western Argentina. In this semi-arid area, the Mendoza river supports irrigated agriculture, hydroelectric power generation, the oil industry, drinking-water supplies, and tourism (Ríos et al., 2022; Toum et al., 2025). The Mendoza river is formed by the confluence of the Cuevas, Tupungato, and Vacas rivers. Of these three main contributors, the Cuevas and Vacas rivers receive meltwater from glaciers and snow from their upper watersheds in the Aconcagua area. Consequently, policies aimed at reducing plastic use and ensuring their proper final disposal at the Aconcagua Provincial Park could mitigate environmental and human health risks in the populated areas of the lower Mendoza river basin in Argentina.

#### Study limitations and future research

While this study provides novel baseline data on microplastic occurrence across an altitudinal gradient on Mount Aconcagua (from ∼4,300 to ∼5,930 m a.s.l.), several limitations should be acknowledged. First, the sample size (N = 16; n = 4 per site) was constrained by the extreme environmental conditions, expedition logistics, and the stringent contamination-control procedures required for high-altitude sampling. Consequently, the statistical analyses and resulting inferences should be interpreted within the context of this preliminary baseline investigation. Second, non-simultaneous sampling among camps was unavoidable because of the progressive ascent of the expedition and the limited weather windows available for safe fieldwork. Nevertheless, all samples were collected during the peak climbing season, when mountaineering activity is typically sustained. Third, because sampling was restricted to a single climbing season, the results represent a temporal snapshot and do not capture the seasonal or interannual variability of microplastic deposition. Finally, although the observed polymer profiles suggest contributions from multiple sources, the relative importance of local inputs versus regional or long-range atmospheric transport could not be assessed because backward air mass trajectory analyses were beyond the scope of the present study. Atmospheric transport and deposition of microplastics in remote high-mountain environments have been successfully investigated using trajectory models such as HYSPLIT (Allen et al., 2019). Future studies should therefore integrate backward air mass trajectory analyses with high-altitude snow monitoring, expand the spatial coverage to include the summit (6,962 m a.s.l.), and establish long-term monitoring programs to provide a more comprehensive assessment of the sources, transport pathways, deposition dynamics, and accumulation of microplastics within Aconcagua Provincial Park.

## Conclusion

This study provides the first evidence of MPs occurrence in snow collected at Mount Aconcagua, the highest peak in the Americas, demonstrating that snow represents a valuable environmental matrix for monitoring MPs in the Andes. Among the sampled sites on Mount Aconcagua, the highest MP concentrations were observed at the Plaza de Mulas base camp (4340 masl), which sustains continuous human presence throughout the austral summer climbing season (November-March). The predominance of microfibers is likely associated with fiber shedding from trekkers’ clothing and climbing equipment, as well as atmospheric transport driven by wind. Overall, the findings suggest that MPs contamination in this high-altitude ecosystem results from a combination of long-range atmospheric deposition and local inputs linked to human outdoor activities. Mountain environments, particularly those at high elevation, present substantial challenges for managing plastic waste generated during expeditions. In this context, policy interventions aimed at reducing plastic consumption and improving waste management practices in mountain regions are critical to preventing local pollution. Effective mitigation will require a multifaceted approach, including the implementation of circular economy strategies, strengthened regulatory frameworks (e.g., restrictions on single-use plastics), and targeted environmental education campaigns to increase awareness among visitors and stakeholders. This baseline assessment contributes novel insights into microplastic pollution at Mount Aconcagua, a heavily visited high-mountain environment, and provides empirical evidence to inform policy development aimed at mitigating plastic pollution and its potential impacts in Mendoza Province.

## Data Availability

The original contributions presented in the study are included in the article/Supplementary Material, further inquiries can be directed to the corresponding author.
