# Supplementary material for: Microplastics at the ‘roof of the Americas’: widespread presence in snow on Mount Aconcagua, Central Andes, Argentina
Source: Front Toxicol. 2026 Oct 8;8:1878370. doi: 10.3389/ftox.2026.1878370 (PMC13687666; doi:10.3389/ftox.2026.1878370)
Supplement: Supplementary file 1 [file Table1.docx]

**Supplementary material**

**Microplastics at the 'Roof of the Americas': Widespread contamination of snow in Mount Aconcagua of the Central Andes, Argentina**

Juan Manuel Ríos^1,*^, Yoshifumi Horie^2^ , Mariano Hugo Masiokas^3^ and Diego Esteban Cargnelutti^1^

^1^ Laboratorio de Ecotoxicología, Instituto de Medicina y Biología Experimental de Cuyo (IMBECU, CCT-CONICET), 5500, Mendoza, Argentina.

^2^ Research Center for Inland Seas (KURCIS), Kobe University, Fukaeminami-machi, Higashinada-

ku, Kobe 658-0022, Japan.

^3^Instituto Argentino de Nivología, Glaciología y Ciencias Ambientales, (IANIGLA), CCT- CONICET, 5500, Mendoza, Argentina.

E-mail: [jmriosrama@gmail.com](mailto:jmriosrama@gmail.com); jrios@mendoza-conicet.gob.ar

*ORCID 0000-0001-7018-1458*

**Running tittle: Microplastics footprint in Mt. Aconcagua**

Supplementary material includes two tables and three figures.

**Table S1**.  Mean, median, and standard error (SE) and range for fiber size detected in the snow of Mount Aconcagua sampling camps.

| Site | Mean  (mm) | Median  (mm) | SE | Range  (mm) |
| --- | --- | --- | --- | --- |
| Plaza de Mulas  (base camp) | 1.05 | 1.25 | 0.35 | 0.2–1.5 |
| Canada  (Camp 1) | 0.91 | 1.00 | 0.23 | 0.3–2 |
| Nido de condores  (Camp 2) | 0.91 | 0.40 | 0.44 | 0.2–5 |
| Berlin  (Camp 3) | 1.04 | 0.80 | 0.17 | 0.3–2 |
| Aconcagua* | 0.98 | 0.80 | 0.14 | 0.2–5 |

* values for the Mt Aconcagua (all sampling camps together).

**Table S2.** Summary of several articles reporting on MPs concentration and characterization in snow samples worldwide, sorted from oldest to newest.

| Site | Altitude  m.a.s.l. | Concentration  MP L^-1^ | Shapes | Polymers* | Reference |
| --- | --- | --- | --- | --- | --- |
| Swiss Alps | 2019 | 9800±6900 | fibers | EVA, Nylon, PE, PP, PS, PC, PPVC, PES, Rubber, Cell. | Bergmann et al., 2019 |
| Forni Ice  Alps Italy | 2580 | 70±32.9 MPs Kg^-1^ | fibers  fragments | PES, PA, PE, PP | Ambrosini et al., 2019 |
| Mt.Everest Nepal | 8440 | 30±11 | fibers  fragments | PES, Acrylic, Nylon, PP | Napper et al., 2020 |
| Antisana  Glacier Ecuador | 5753 | 131 | fibers fragments  spheres  film | PU, PE, PA, PES, HDPE. | Cabrera et al., 2022 |
| Carnic Alps Italy | 1872 | 0.11±0.19 | fragments | PET | Pastorino et al., 2021 |
| Western Alps Italy | 2500 | 4.9±2.5 | fibers  fragments | PE, PET, PES, PC, PP, PU | Parolini et al., 2021 |
| Tibetan plateau | 4460–5600 | n.d. | fibers  fragments | PA, PE, rubbers, PET, PS, PC,  PMMA | Zhang et al., 2021 |
| Sonnblick  Austria | 3106 | 46.5 mg/L | fibers  spheres | PET, PP, PS, PE | Kau et al., 2021 |
| Vatnajökull Iceland | 1400 | n.d. | fibers  fragments | PU, PVC, PA, ABS, Cell | Stefansson et al., 2021 |
| Barnaul  Russia | 180 | 27–595 | fibers fragments  foam  film | n.d. | Chernykh et al., 2022 |
| Tabriz  Iran | 1350 | 12–20 | fibers fragments  spheres  film | Nylon, Cell., PP, PES, PET, EVA, PS, PVC, PE | Abbasi et al., 2022 |
| Ross Island  Antarctica | n.d. | 29.4±4.7 | fibers fragments  film | PET, PMMA, PVC, PA, PE, cell | Aves et al., 2022 |
| Tenerife Spain | 2150–3200 | 51±72  188±164 | fibers fragments  film | Cell., PES, Acrylic, Nylon, PP, Azlon | Villanova-Solano  et al., 2023 |
| Inner Mongolian  plateau | 300 | 68±10  199±22 | fibers fragments  foam | PE, PA, PES, PS, PVC, PET | Yu et al., 2023 |
| Hokkaido  Japan | 5–1143 | 150  4200 | fragments | PE, rubber, PA, PVC, PMMA,  rayon, PU, EVA | Ohno and Lizuka,  2023 |
| Ankara  Turkey | 5–938 | 59 | fibers fragments  film | PE, PS, PP, PET, PVC, Nylon | Babaei et al., 2023 |
| Conness glacier  USA | 3292 | 539 | fibers fragments | PA, PS, Polyvinylester, PET, polyolein | Karapetrova et al.,  2024 |
| Qom  Iran | 928 | 23.8±15.8  1.26±1.16 | fibers rubbers | PET, nylon, PP, PE, PS, PVC | Ranjdoost et al.,  2024 |
| Mt. Terminllo | 2217 | 74 | fibers fragments  film | PA, PP, PE, PET, PES, Cell., PA, PTFE, ABS | Pietrelli et al. 2025 |
| Western [Siberia](https://www.sciencedirect.com/topics/earth-and-planetary-sciences/siberia), Russia | 252 | 2817 MP m^−2^ | fibers fragments | [PET](https://www.sciencedirect.com/topics/agricultural-and-biological-sciences/polyethylene-terephthalate), [PA](https://www.sciencedirect.com/topics/agricultural-and-biological-sciences/polyamide), [PC](https://www.sciencedirect.com/topics/earth-and-planetary-sciences/polycarbonate), [PP](https://www.sciencedirect.com/topics/earth-and-planetary-sciences/polypropylene), Rayon | Frank et al. 2025 |
| Mt. Aconcagua  Argentina | 6921 | 38±11 | fibers fragments | Polyester, PE, Epoxy, Polyvinychloride, polyarylate, polystirene-co-acrylamide | This study |

*PP* = polypropylene, *PA* = polyamide, *PE* = polyethylene, *PTFE* = polytetraluorethylene, *PES* = polyester, *PET* = polyethylene terephthalate, *HDPE =* high-density polyethylene, *ABS* = Acrylonitrile–Butadiene–Styrene, *PS* = Polystyrene, *PVC* = Polyvinylchloride, *PU* = Polyurethan, *EVA* = Ethylenevinlacetate, *PMMA* = Polymethylmethacrylate, *Cell* = Cellulose based. n.d. = no determined.

**Fig. S1.** Percentage contribution of MPs shapes present in the snow samples of Mt. Aconcagua

**Fig. S2.** Percentage contribution of fiber color present in the snow samples according to sampling camps of Mt. Aconcagua

**Fig. S3.** Percentage contribution of polymer types identified in the snow samples of Mt. Aconcagua in all sampling site (camps) together.

**References**

Abbasi, S., Alirezazadeh, M., Razeghi, N., Rezaei, M., Pourmahmood, H., Dehbandi, R., ... and Turner, A. (2022). Microplastics captured by snowfall: A study in Northern Iran. *Sci. Total Environ.* *822*, 153451. [doi: 10.1016/j.scitotenv.2022.153451](https://doi.org/10.1016/j.scitotenv.2022.153451)

Ambrosini, R., Azzoni, R. S., Pittino, F., Diolaiuti, G., Franzetti, A., and Parolini, M. (2019). First evidence of microplastic contamination in the supraglacial debris of an alpine glacier. *Environ. Pollut.* *253*, 297-301. doi: 10.1016/j.envpol.2019.07.005

Aves, A. R., Revell, L. E., Gaw, S., Ruffell, H., Schuddeboom, A., Wotherspoon, N. E., ... and McDonald, A. J. (2022). First evidence of microplastics in Antarctic snow.  *Cryosphere*, *16*(6), 2127-2145. doi.org/10.5194/tc-16-2127-2022

Babaei, P., Nikravan, A., Meral, A., Kibar, B., and Güllü, G. (2023). A first assessment of microplastic contamination in the snow of Ankara, Turkey. *Environ. Sci. Pollut. Res. 30*(47), 103690-103702. doi: 10.1007/s11356-023-29594-3

Bergmann, M., Mützel, S., Primpke, S., Tekman, M. B., Trachsel, J., and Gerdts, G. (2019). White and wonderful? Microplastics prevail in snow from the Alps to the Arctic. *Sci. Adv.* *5*(8), eaax1157. [doi: 10.1126/sciadv.aax1157](https://doi.org/10.1126/sciadv.aax1157)

Cabrera, M., Moulatlet, G. M., Valencia, B. G., Maisincho, L., Rodríguez-Barroso, R., Albendín, G., ... and Capparelli, M. V. (2022). Microplastics in a tropical Andean Glacier: A transportation process across the Amazon basin?. *Sci. Total Environ.* *805*, 150334.

doi: 10.1016/j.scitotenv.2021.150334.

Chernykh, D. V., Biryukov, R. Y., Kuryatnikova, N. A., and Malygina, N. S. (2022). Microplastics in the snow cover of urban landscapes: A case study of Barnaul. *Geogr. Nat. Resour.* *43*(Suppl 1), S44-S49. doi:[10.1134/S1875372822050080](https://doi.org/10.1134/S1875372822050080)

Frank, Y. A., Tatsii, D., Rednikin, A. R., Plach, A., Rakhmatullina, S. N., Vorobiev, D. S., and Stohl, A. (2025). It is snowing microplastics in Western Siberia. *Environ. Pollut.* *364*, 125293. doi:[10.1016/j.envpol.2024.125293](https://doi.org/10.1016/j.envpol.2024.125293)

Karapetrova, A., Cowger, W., Michell, A., Braun, A., Bair, E., Gray, A., and Gan, J. (2024). Exploring microplastic distribution in Western North American snow. *J. Hazard. Mater.* *480*, 136126. [doi.org/10.1016/j.jhazmat.2024.136126](https://doi.org/10.1016/j.jhazmat.2024.136126)

Kau, D., Materić, D., Holzinger, R., Baumann-Stanzer, K., Schauer, G., and Kasper-Giebl, A. (2024). Fine micro-and nanoplastics concentrations in particulate matter samples from the high alpine site Sonnblick, Austria. *Chemosphere*, *352*, 141410. [doi.org/10.1016/j.chemosphere.2024.141410](https://doi.org/10.1016/j.chemosphere.2024.141410)

Napper, I. E., Davies, B. F., Clifford, H., Elvin, S., Koldewey, H. J., Mayewski, P. A., ... and Thompson, R. C. (2020). Reaching new heights in plastic pollution—preliminary findings of microplastics on Mount Everest. *One Earth*, *3*(5), 621-630. doi:[10.1016/j.oneear.2020.10.020](https://doi.org/10.1016/j.oneear.2020.10.020)

Ohno, H., and Iizuka, Y. (2023). *Microplastics in snow from protected areas in Hokkaido, the northern island of Japan. Sci. Rep. 13 (1), 9942*. doi.org/10.1038/s41598-023-37049-5

Parolini, M., Antonioli, D., Borgogno, F., Gibellino, M. C., Fresta, J., Albonico, C., ... and Cavallo, R. (2021). Microplastic contamination in snow from Western Italian Alps. *Int. J. Environ. Res. Public Health.* *18*(2), 768. [doi.org/10.3390/ijerph18020768](https://doi.org/10.3390/ijerph18020768)

Pastorino, P., Pizzul, E., Bertoli, M., Anselmi, S., Kušće, M., Menconi, V., ... and Renzi, M. (2021). First insights into plastic and microplastic occurrence in biotic and abiotic compartments, and snow from a high-mountain lake (Carnic Alps). *Chemosphere*, *265*, 129121.

doi: 10.1016/j.chemosphere.2020.129121

Pietrelli, L., Menegoni, P., Millesimi, I., Sighicelli, M., Baldi, M., and Schirone, B. (2025). Microplastics in the Mount Terminillo (Rieti, Italy) Snow’s. *Water, Air, Soil Pollut. 236*(7), 402. doi:[10.1007/s11270-025-07917-z](https://doi.org/10.1007/s11270-025-07917-z?urlappend=%3Futm_source%3Dresearchgate.net%26utm_medium%3Darticle)

Ranjdoost, F., Abbasi, S., Asadi-Ghalhari, M., Baghani, A. N., Fouladi-Fard, R., Sorooshian, A., and Ghaffari, M. E. (2024). On the nature and sources of microplastics (MPs) and microrubbers (MRs) in urban snow. *J. Environ. Manage. 370, 122851.*

doi: 10.1016/j.jenvman.2024.122851

Stefánsson, H., Peternell, M., Konrad-Schmolke, M., Hannesdóttir, H., Ásbjörnsson, E. J., and Sturkell, E. (2021). Microplastics in glaciers: first results from the Vatnajökull ice cap. *Sustainability*, *13*(8), 4183. doi:[10.3390/su13084183](https://doi.org/10.3390/su13084183?urlappend=%3Futm_source%3Dresearchgate.net%26utm_medium%3Darticle)

Villanova-Solano, C., Hernández-Sánchez, C., Díaz-Peña, F. J., González-Sálamo, J., González-Pleiter, M., and Hernández-Borges, J. (2023). Microplastics in snow of a high mountain national park: El Teide, Tenerife (Canary Islands, Spain). *Sci. Total Environ.* *873*, 162276. [doi.org/10.1016/j.scitotenv.2023.162276](https://doi.org/10.1016/j.scitotenv.2023.162276)

Yu, H., Shao, J., Jia, H., Gang, D., Ma, B., and Hu, C. (2024). Characteristics and influencing factors of microplastics in snow in the inner Mongolian plateau, china. *Engineering*, *37*, 69-77. [doi.org/10.1016/j.eng.2023.02.007](https://doi.org/10.1016/j.eng.2023.02.007)

Zhang, Y., Gao, T., Kang, S., Allen, S., Luo, X., and Allen, D. (2021). Microplastics in glaciers of the Tibetan Plateau: Evidence for the long-range transport of microplastics. *Sci. Total Environ.* 1:758:143634. doi: 10.1016/j.scitotenv.2020
